# Dopamine Transporter Density in *de novo* Parkinson’s Disease Does Not Relate to the Development of Levodopa-Induced Dyskinesias

**Published:** 2019-06-05

**Authors:** Andreas-Antonios Roussakis, Marta Gennaro, Nicholas P. Lao-Kaim, David Towey, Paola Piccini

**Affiliations:** 1 Neurology Imaging Unit, Imperial College London, UK; 2 Radiological Sciences Unit, Imperial Healthcare NHS Trust, UK

**Keywords:** Parkinson's disease, dyskinesias, de novo, single photon emission computed tomography, imaging

## Abstract

**Background:**

In Parkinson’s disease (PD), the onset of levodopa-induced dyskinesias (LIDs) is difficult to predict. This study examines whether dopamine transporter (DAT)-specific SPECT imaging in *de novo* PD relates to later development of LIDs.

**Methods:**

42 *de novo* unilateral PD participants received DAT-specific SPECT imaging with ^123^I-FP-CIT at time of diagnosis. At five years post-diagnosis, all PD patients were clinically evaluated and divided into two groups based on whether they had or had not developed LIDs. Fourteen gender- and age-matched healthy volunteers undertook ^123^I-FP-CIT SPECT imaging and were included as controls. A semi-quantification approach was used for the ^123^I-FP-CIT data using the occipital cortex as the reference region. We calculated specific binding ratios (SBR) for the caudate and putamen (posterior and anterior putaminal subregions). In parallel, we analysed our ^123^I-FP-CIT dataset with a voxel-based analysis approach.

**Results:**

PD patients had significantly lower striatal ^123^I-FP-CIT SBR values in comparison to controls (*p*<0.001). After five years, dyskinetic patients (N=10) were taking higher daily doses of dopaminergic medication (*p*<0.001) and had more severe disease (difference in Hoehn & Yahr staging scores *p*<0.05) as compared to the non-dyskinetic group (N=32). At the time of diagnosis, ^123^I-FP-CIT SBR values were not statistically different between the two groups for all striatal regions (*p*>0.05). SPM voxel-based analysis did not show a statistically significant difference between the two groups (*p*>0.05).

**Conclusion:**

^123^I-FP-CIT SPECT imaging, performed at diagnosis in *de novo* early-stage PD could not differentiate patients who will develop LIDs within five years from those who will not.

## Introduction

Long-term treatment plans in Parkinson’s disease (PD) are commonly complicated by the development of levodopa-induced dyskinesias (LIDs). Epidemiology studies have linked LIDs to the duration of levodopa treatment [1] and longer disease duration; approximately 60% of patients treated with levodopa for five years develop LIDs [2,3]. While the underlying mechanisms of LIDs are complex and not entirely understood, evidence from clinical imaging studies [4–9] implicate presynaptic neuronal mechanisms in misregulation of exogenous levodopa within the striatum.

Presynaptic imaging of the dopamine transporter (DAT) using [123I]N-w-fluoropropyl-2β-carbomethoxy-3β-(4-iodophenyl)nortropane (^123^I-FP-CIT) SPECT [10] differentiates individuals with PD from normal controls quite reliably and is commonly used for diagnostic purposes. A recent study performed in Seoul, Korea using ^18^F-FP-CIT PET showed that putaminal DAT density in PD was useful to predict later expression of LIDs [11]. At initial evaluation, PD patients who developed LIDs had already significantly lower putaminal DAT density than those patients who did not become dyskinetic. To our knowledge, the possible development of LIDs has not been studied with ^123^I-FP-CIT SPECT (which in clinical practice is more available than ^18^F-FP-CIT PET) in early, *de novo* PD patients, who represent the majority of new patients within outpatient clinics.

The present study aims to assess whether ^123^I-FP-CIT SPECT in *de novo* PD can differentiate patients who develop LIDs from those who do not become dyskinetic five years after initial diagnosis, and whether semi-quantification of ^123^I-FP-CIT SPECT data can be useful in this regard for prospective treatment management.

## Methods

### Participants

The study was conducted in accordance with the Declaration of Helsinki following approval by the NRES West London Research Ethics Committee and all appropriate regulatory approvals. Recruitment of fourteen healthy volunteers as controls was conducted through publicity posters. All subjects provided their consent in writing. 73 patients who were clinically diagnosed with Parkinsonism, had abnormal ^123^I-FP-CIT SPECT imaging, and fulfilled the Queen Square Brain Bank diagnostic criteria for idiopathic PD [12] were selected from the movement disorders clinics of the Imperial College Healthcare NHS Trust in London. Of those, we included only the PD patients who were drug-naïve and had Hoehn & Yahr stage 1 at the time of ^123^I-FP-CIT SPECT imaging. None of the above patients had concomitant neuro-psychiatric disease.

Based on evidence from the epidemiology studies about the usual onset of LIDs [2,3], we assessed the presence/absence of LIDs at five years from diagnosis. PD patients who after five years from diagnosis were still drug-naïve or for whom we had missing data or unresolved queries were excluded from the study. From a clinical point of view, PD patients were reviewed by the same clinical team at least every six months and were prescribed levodopa and other dopaminergic medicines based upon their individual needs.

### Clinical data

Clinical data for the PD patients included disease duration from clinical diagnosis (DD_diagn_), medication history, PD-related asymmetry, Hoehn and Yahr (H&Y) staging [13], presence/absence of LIDs, and daily levodopa equivalent doses (LEDs), as described previously [9]. We calculated LEDs based on individual prescriptions at five years after diagnosis. At enrolment, PD patients were matched for disease progression, by selecting the patients who were at H&Y stage 1 at the time of SPECT imaging. We divided patients into two groups: those who had experienced LIDs five years after the diagnosis (PD LIDs, N=10) and those who had no recorded history for dyskinesia (PD non-LIDs, N=32). Presence/absence of LIDs was sought at each clinical visit and acurately recorded by the same clinical team for all enrolled PD patients.

### Scanning procedures

All controls and PD patients had one ^123^I-FP-CIT SPECT scan in accordance with the clinical protocol of the Nuclear Medicine department. Briefly, ^123^I-FP-CIT was injected intravenously as a single bolus injection (mean activity dose of 185 MBq). ^123^I-FP-CIT SPECT acquisition started 180 minutes after injection and acquired while participants were at rest for 45 minutes (acquisition parameters: 128 views with 128x128 matrix and 1.45 zoom with 30 seconds per view in step-and-shoot mode; 15% energy window centred on the 159 keV photopeak of ^123^I; 2 million total counts). PD patients also had a 1.5 Tesla T1-weighted MRI scan to exclude ischaemic disease in the basal ganglia.

## ^123^I-FP-CIT SPECT analysis

### Semi-quantification

Acquired SPECT data were transferred to the HERMES-workstation of the Nuclear Medicine department. Reconstructed tomographic data were analysed using BRASS^™^ (HERMES medical solutions) [14,15]. The software uses automatic image registration to align the examinees image to the EARL.db template, composed of the scans of twenty independent healthy individuals. SPECT images were reconstructed using the default ordered subset expectation maximization algorithm that incorporates corrections for attenuation (Chang method; attenuation coefficient μ=0.12 cm^-1^; as in [16]), scatter (Monte Carlo simulation; as in [15,17]) and camera and collimator resolution recovery using Hybrid Recon^™^ (HERMES medical solutions). SPECT data were corrected for camera-specific image properties as defined by respective phantom measurements.

The reconstructed SPECT images were smoothed using a 3D Gaussian image filter (full width at half maximum = 7 mm). During automatic fitting with BRASS^™^, the normalised mutual information function was used to determine the similarity of the realigned image to the template [18], as previously recommended [19–21]. Following automatic fitting, predefined striatal volumes of interest (VOIs) were applied. All scans were inspected visually and, where necessary, manually realigned to fit to the predefined template. SPECT studies with excessive motion were discarded. A regional reference volume centred on the occipital cortex was used to estimate nonspecific binding. This reference volume was then used to scale VOI counts/voxel such that resulting values represented the specific to nonspecific binding ratio (SBR), calculated as: SBR = (VOI - Reference) / (Reference). SBR values were calculated for the caudate, putamen, anterior and posterior putamen for the most and least affected hemispheres for each subject. Averaged SBR_VOI_ values were calculated per individual as the mean SBR_VOI_ values across both hemispheres [i.e. (left SBR_VOI_ + right SBR_VOI_)/2].

### Voxel-based analysis

Reconstructed SPECT images were converted from DICOM to Analyze format using MANGO (UTHSCSA). For the purposes of this study, the right hemisphere was selected to represent the most affected side of the brain, defined as the hemisphere contralateral to the clinically symptomatic body side. Subsequently, the left hemisphere represents the least affected side of the brain. As such, individuals who first displayed symptoms on the right of the body had their scans flipped (using MANGO). Statistical voxel-based analysis was performed using SPM_12_ (UCL) implemented in Matlab, Version R2017b (MathWorks). SPECT images were then spatially normalised to Montreal Neurological Institute stereotactic space using a validated ^123^I-FP-CIT SPECT template [22]. Normalised data were smoothed with a Gaussian kernel of 6 × 6 × 6 mm at full-width at half-maximum, to accommodate interindividual variability and improve the signal-to-noise ratio. An occipital lobe mask was created using the WFU-PickAtlas SPM toolbox [23]. Mean voxel values in the occipital lobe were extracted using the MarsBaR SPM toolbox [24]. Global counts were then normalised by proportional scaling with the occipital lobe to remove confounding effects. Groups were compared on a voxel-by-voxel basis using a two-sample t-test in SPM_12_. The voxel-based analysis was limited to the striatum using an explicit mask created using WFU-PickAtlas. Differences between all PD patients and controls were assessed at a *p*<0.05 voxel height threshold corrected for family-wise error. Differences in the scans between the PD LIDs and PD non-LIDs groups were tested at a *p*<0.05 voxel height threshold FWE corrected. Age and gender were included as nuisance covariates.

### Statistical analyses

Statistical analyses on clinical data and SBR values were performed using SPSS, Version 25 (IBM). Graph illustrations were performed using Prism, Version 6 (GraphPad Software). Homogeneity and normal distribution were tested with Bartlett’s and Kolmogorov-Smirnov tests. Details of each statistical test are documented in the legends of the tables and figure. The significance level was set at α=0.05.

## Results

### Clinical findings

The time between ^123^I-FP-CIT SPECT imaging and diagnosis of idiopathic PD was 1.11 ± 1.51 years. By five years post-diagnosis, all PD patients (N=42; 27M:15F) had been on pharmaceutical medication for PD for a minimum of two years. By that time, 10 PD patients (6M:4F) had developed LIDs and 32 PD patients (21M:11F) had never experienced LIDs in the clinic not at home to report so to the clinical team. The PD LIDs group were significantly younger as compared to the PD non-LIDs group (*p*<0.05). At five years post-diagnosis, the PD non-LIDs group had significantly higher H&Y scores (*p*<0.05) and were taking higher LEDs (*p*<0.001) (Table 1).

### ^123^I-FP-CIT SPECT Imaging

#### Semi-quantification

**Figure 1:**
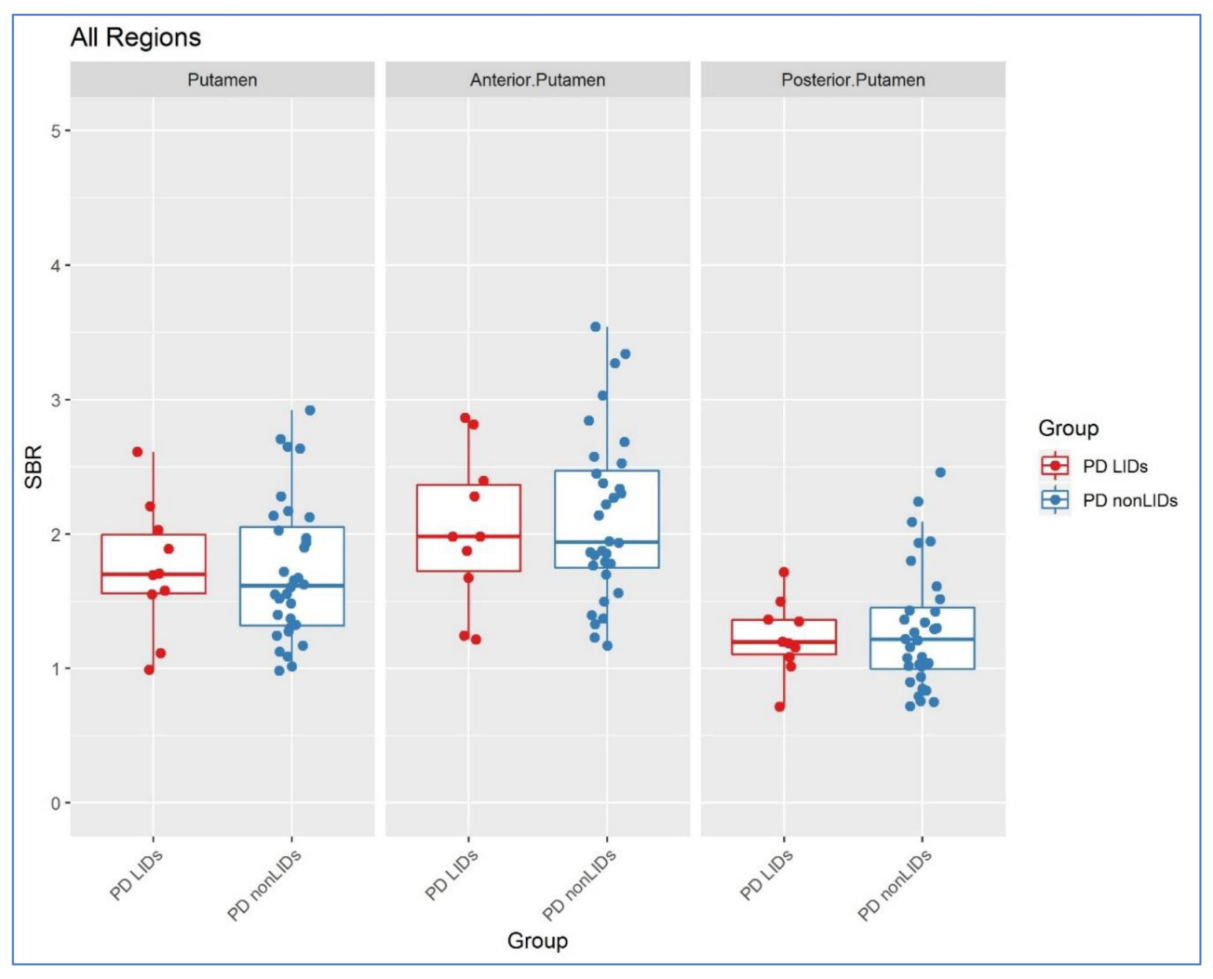
Scatter plots of ^123^I–FP–CIT SBR values in the puatmen, anterior putamen, and posterior putamen between the two PD groups

As expected, the PD group as a whole had significantly lower ^123^I-FP-CIT SBR values in all studied VOIs, as compared to controls (*p*<0.001). However, ^123^I-FP-CIT SBR values for the PD LIDs group were not statistically different to values for the PD non-LIDs group in the putamen, anterior putamen, posterior putamen or caudate (*p*>0.05). This was true for SBR values on the most and least affected sides as well as those averaged between hemispheres (Figure 1, Tables 2 & 3).

### Voxel-based analysis

Voxel-based analysis showed a significant reduction in striatal ^123^I-FP-CIT specific-to-nonspecific binding in the entire PD cohort as compared to controls. This reduction was statistically significant in both hemispheres and greatest in the putamen of the most affected hemisphere (*p*<0.001). We did not find any statistically significant difference in striatal ^123^I-FP-CIT specific-to-nonspecific binding between the PD LIDs and PD non-LIDs groups, even after the voxel height threshold was set leniently at *p*<0.05 uncorrected.

## Discussion

In this study, we investigated in *de novo* PD whether ^123^I-FP-CIT SPECT imaging at diagnosis is related to the occurrence of future LIDs. The primary aim of the study is of clinical importance, because at present, there is no robust evidence to guide clinicians on how to best design a long-term therapeutic plan and delay (if possible) the onset of peak dose dyskinesias. We found that, striatal DAT density, evaluated using ^123^I-FP-CIT SPECT at diagnosis, does not differentiate between the patients who developed LIDs from those who did not become dyskinetic over the five-year period. This finding is at variance with a previous study reporting a predictive role of DAT-specific imaging in the development of dyskinesias [11].

The imaging study by Hong and colleagues [11] found that PD patients who later developed LIDs had more pronounced striatal DAT deficits than the patients who did not develop LIDs. The study, however, included PD patients who at baseline were already highly variable from a severity point of view; disease severity [Unified Parkinson’s Disease Rating Scale (UPDRS) scores] recorded in “off” state, at diagnosis or within 6 months from diagnosis at the time of the scan was statistically different between the two groups (28.4 ± 10.7 versus 19.3 ± 9.4, p<0.001). It is possible that the patients who at baseline were more advanced from a clinical point of view, were the ones who developed LIDs earlier. Hence, in that study, the statistical difference in disease severity at the time of scan between the two PD groups was the driving factor for the prediction of dyskinesias.

To avoid the above bias, in our study design, we deliberately selected *de novo* PD patients who at baseline were at the same stage of disease severity (H&Y stage 1). In this homogenous cohort of PD, ^123^I-FP-CIT SBR values were quite similar for the two groups, hence DAT-specific SPECT at diagnosis could not predict the development of LIDs. From a clinical progression point of view, the two PD groups of our cohort, though they were matched for H&Y staging at diagnosis, progressed differently over time. This is indicated by the fact, that they were taking significantly different doses of levodopa and had a statistical difference in their H&Y scores at the end of the five-year period. It should be noted that the PD patients who developed LIDs five years from diagnosis were younger in comparison to those who did not develop LIDs. This is not confounding the interpretation of these results, but it is in line with the hypothesis that younger at onset patients are prone to develop LIDs earlier [27].

LIDs may be unnoticeable by individual patients at the time they experience them for the first time, or they may be incorporated into socially acceptable movements. Given the evidence from epidemiological studies that the vast majority of PD patients develop LIDs during the first five years of the disease [2,3], we found appropriate to not to estimate the actual onset of LIDs but to record the presence/absence of LIDs five years post-diagnosis.

In conclusion, our findings indicate that striatal DAT density as measured by using ^123^I-FP-CIT SPECT in *de novo* PD does not relate to the occurrence of future LIDs.

## Figures and Tables

**Table 1:** Demographics and clinical characteristics

	Controls	PD All	*p* values	PD non–LIDs	PD LIDs	*p* values
No. of participants	14	42	-	32	10	-
^a^Gender	7M:7F	27M:15F ^ns^	0.343	21M:11F	6M:4F ^ns^	0.746
**At the time of ^123^I–FP–CIT SPECT scan**						
bAge (years)	62.74 ± 9.18	63.95 ± 10.70 ^ns^	0.706	65.96 ± 9.25	57.49 ± 12.87*	0.027
bDD_diagn_ (years)	-	1.11 ± 1.51	-	1.17 ± 1.65	0.91 ± 0.91 ^ns^	0.650
H&Y stage (1-5)	-	1	-	1	1	-
DailyLED_Total_ (mg)	-	-	-	-	-	-
**5 years post clinical diagnosis**						
bAge	-	67.20 ± 11.04	-	68.74 ± 10.05	62.28 ± 13.12^ns^	0.107
DD_diagn_	-	5	-	5	5	-
cH&Y stage	-	2.12 ± 0.55	-	2.00 ± 0.47	2.50 ± 0.59*	0.026
bDaily LED_Total_	-	410.10 ± 234.08	-	355.47 ± 227.41	728.90 ± 147.42***	<0.001
bDaily LED_Ldopa_	-	332.95 ± 223.38	-	254.22 ± 176.93	584.90 ± 164.06***	<0.001
bDaily LED_Dag_	-	111.43 ± 155.26	-	101.25 ± 138.95	144.00 ± 204.35 ^ns^	0.454

Data represent mean values ± 1 SD

a Comparison with chi–squared (χ^2^) test

b Independent *t*-test (two-tailed, equal variances assumed)

c Mann–Whitney U test (calculated by multiplying the one-tailed significance level by two)

ns: no statistically significant difference

* statistical significance *p*<0.05

*** statistical significance *p*<0.001

**Table 2:** ^123^I-FP-CIT SBR values (averaged)

	Controls	PD patients				*p* values
		PD All	*p* values	PD non–LIDs	PD LIDs	
No. of participants	14	42		32	10	
^a^Caudate	3.38 ± 0.40	2.70 ± 0.63***	<0.001	2.72 ± 0.56	2.65 ± 0.86 ^ns^	0.769
^a^Putamen	3.03 ± 0.33	1.73 ± 0.51***	<0.001	1.72 ± 0.52	1.74 ± 0.48 ^ns^	0.941
^a^Anterior Putamen	3.42 ± 0.34	2.10 ± 0.61***	<0.001	2.12 ± 0.63	2.03 ± 0.57 ^ns^	0.698
^a^Posterior Putamen	2.61 ± 0.36	1.28 ± 0.42***	<0.001	1.29 ± 0.46	1.23 ± 0.28 ^ns^	0.669

Data represent mean values ± 1 SD

a Independent *t*-test (two-tailed, equal variances assumed)

ns: no statistically significant difference

*** statistical significance *p*<0.001

**Table 3:** ^123^I-FP-CIT SBR values (between-group comparison for most and least affected sides)

	most affected side			least affected side		
	PD non–LIDs	PD LIDs		PD non–LIDs	PD LIDs	
No. of participants	32	10		32	10	
			***p* values**			***p* values**
^a^Caudate	2.53 ± 0.52	2.50 ± 0.85 ^ns^	0.876	2.90 ± 0.65	2.80 ± 0.88 ^ns^	0.699
^aPutamen^	1.54 ± 0.47	1.54 ± 0.47 ^ns^	0.980	1.90 ± 0.61	1.93 ± 0.54 ^ns^	0.882
^aAnterior Putamen^	1.93 ± 0.64	1.83 ± 0.51 ^ns^	0.651	2.31 ± 0.69	2.23 ± 0.72 ^ns^	0.774
^aPosterior Putamen^	1.12 ± 0.35	1.03 ± 0.26 ^ns^	0.460	1.47 ± 0.62	1.42 ± 0.32 ^ns^	0.836

Data represent mean values ± 1 SD

a Independent *t*-test (two-tailed, equal variances assumed)

ns: no statistically significant difference

## Abbreviations

^123^I-FP-CIT: [^123^I]N-w-FluoroPropyl-2β-Carbomethoxy-3β-(4-Iodophenyl)norTropane
DAT: Dopamine Transporter
DD_diagn_: Disease Duration from clinical diagnosis
H&Y: Hoehn and Yahr
LEDs: Levodopa Equivalent Doses
LIDs: Levodopa-Induced Dyskinesias
PD: Parkinson’s Disease
SBR: Specific Binding Ratio
UPDRS: Unified Parkinson’s Disease Rating Scale
VOI: Volume of Interest
